# Multi-family Therapy for Eating Disorders Across the Lifespan

**DOI:** 10.1007/s11920-024-01504-5

**Published:** 2024-05-06

**Authors:** Julian Baudinet, Ivan Eisler

**Affiliations:** 1 Maudsley Centre for Child and Adolescent Eating Disorders (MCCAED), Maudsley Hospital, De Crespigny Park, Denmark Hill, London, SE5 8AZ UK; 2 Centre for Research in Eating and Weight Disorders (CREW), Institute of Psychiatry, Psychology & Neuroscience (IoPPN), King’s College London, De Crespigny Park, Denmark Hill, London, SE5 8AF UK

**Keywords:** Maudsley family therapy, Multi-family therapy, FT-AN, FT-BN, Family based treatment, FBT, Anorexia nervosa, Bulimia nervosa, Group therapy

## Abstract

**Purpose of Review:**

This review aims to report on recent evidence for multi-family therapy for eating disorders (MFT) across the lifespan. It is a narrative update of recent systematic, scoping and meta-analytic reviews.

**Recent Findings:**

There has been a recent increase in published theoretical, quantitative and qualitative reports on MFT in the past few years. Recent and emerging data continues to confirm MFT can support eating disorder symptom improvement and weight gain, for those who may need to, for people across the lifespan. It has also been associated with improved comorbid psychiatric symptoms, self-esteem and quality of life. Data are also emerging regarding possible predictors, moderators and mediators of MFT outcomes, as well as qualitative data on perceived change processes. These data suggest families with fewer positive caregiving experiences at the start of treatment may particularly benefit from the MFT context. Additionally, early change in family functioning within MFT may lead to improved outcomes at end of treatment.

**Summary:**

MFT is a useful adjunctive treatment across the lifespan for people with eating disorders. It helps to promote change in eating disorder and related difficulties. It has also been shown to support and promote broader family and caregiver functioning.

## Introduction

Multi-family therapy (MFT) is increasingly being implemented in eating disorder services internationally [1•]. MFT models differ significantly depending on the country and service context. What they all have in common is that they are (a) group-based, (b) involve several families working together as part of treatment with the support of a clinical team and, (c) in the context of eating disorders, are most commonly delivered in blocks of several hours or full days of treatment [2, 3]. MFT has been described in the mental health literature since the 1960s. The earliest groups described were for people struggling with schizophrenia [4, 5], substance misuse [6] and depression [7, 8]. Nowadays, MFT is used internationally to support people with a range of presentations across the lifespan including eating disorders, mood disorders, schizophrenia, addiction and medical illnesses [9].

MFT for eating disorders was first described for young adults with bulimia nervosa [10] and anorexia nervosa [11] in the 1980s. Adolescent-specific MFT models emerged in the 1990s, initially from teams in Germany [12, 13] and the UK [14]. These MFT models integrated the theoretical concepts of eating disorder focused family therapy [15–18] with more general concepts of MFT [14, 19, 20].

The overarching aim of current MFT models are to speed up the recovery process by offering brief, intensive support in the early stages of treatment when families are feeling most in need of urgent support. MFT differs from other intensive outpatient treatment options (e.g., day programmes, partial hospitalisation programmes, intensive outpatient treatment, etc.) by being entirely group-based and typically adjunctive to other therapeutic interventions [21]. By bringing multiple families together, MFT provides an opportunity for them to learn from each other and share experiences with a wider range of people, including others with lived experience [13, 22]. This is described as helping to reduce stigma and a sense of isolation [23, 24]. The MFT milieu has also been described as a “hot house” learning environment in which it is safe to try out new behaviours and express emotions with increased support [19]. The collaborative environment of MFT may also help to highlight and address any deleterious staff-patient dynamics that can otherwise hamper treatment engagement and progress [13].

Several systematic, scoping and meta-analytic reviews have been published on MFT across the lifespan in recent years [1, 25, 26]. MFT Manuals have been produced in English [27, 28, 29•, 30], French [31], Swedish [32, 33], and German [34].

Typically, MFT is offered in an outpatient context, although day- and inpatient models have also been described. The briefest MFT models provide three [35] or five [28, 36–39] consecutive days of MFT offered as a stand-alone intervention. The latter five-day model has been manualised by a team at the University of California, San Diego, and named Temperament-Based Therapy with Support (TBT-S [28]). Other models, such as the Maudsley model, offer six to ten days of MFT spread across 6–12 months [29•, 40]. Others offer up to 20 days or more of MFT [41–43•] spread across a period of 12 months or more. Inpatient MFT models tend to be more heterogeneous. Those described in the literature range from the aforementioned three and five day models to eight-week [44], 26-week [45] and 12-month models [46].

The majority of programmes described in this paper are outpatient child and adolescent MFT models for anorexia nervosa (MFT-AN), although the number of adult studies is growing. Only recently have MFT models specifically for bulimia nervosa (MFT-BN) been developed [47, 48]. Several services offer MFT for mixed eating disorder groups [36, 46, 49•]. During child and adolescent MFT, parents and siblings are typically invited to attend, whereas adult models may also include partners, other significant people in the participant’s life, and/or parents/caregivers.

This review aims to narratively synthesise key MFT findings and provide an update on new quantitative and qualitative data published since our most recent systematic scoping review [1]. While the full systematic scoping review process was not repeated, the same search terms and strategy were implemented using the same databases (PsycInfo, Medline, Embase, CENTRAL) with the date range set for April 2021 to the present.

### Outpatient Multi-family Therapy Outcomes

Regardless of age or MFT model, the pattern of findings is relatively similar across studies. One outpatient MFT-AN randomized controlled trial (RCT) has been published to date (*N* = 169, age range 12–20 years) [50•], although two other RCT protocols have been reported [51, 52], suggesting more is to follow. Eisler et al. [50•] randomised participants to 12 months of single-family therapy alone or single-family therapy with the addition of 10 days of adjunctive MFT-AN spread across treatment. Both groups reported significant improvements in weight, eating disorder psychopathology and mood, as well as parent-rated negative aspects of caregiving. Participants who received MFT-AN were significantly more likely to have a better global outcome, using the Morgan Russel outcome criteria [53], compared to those who received single-family therapy alone. At six-month follow-up (18 months post-randomisation) this difference was no longer significant, however, weight was significantly higher for those who received MFT-AN compared to single-family therapy [50•].

Findings from uncontrolled comparison studies and case series’ confirm MFT is associated with weight gain for those who are underweight [43•, 49•, 50•, 54–57] and a reduction in eating disorder symptoms [3, 43•, 49•, 50•, 56, 57].

Meta-analytic review findings suggest a large effect size for weight (*n* = 8 studies) and medium effect size for self-reported eating disorder symptoms (*n* = 4 studies) from baseline to post-MFT intervention, although no differences were reported between MFT and comparison interventions [26]. When subgroup analyses of weight outcomes were assessed by age, adolescent MFT was associated with significant weight gain with a large effect (standard mean difference = 1.15, 95% CI = 0.94, 1.36), but adult MFT was not (standard mean difference = 0.18, 95% CI = − 0.09, 0.45). It is worth noting that only three adult studies [39, 45, 46] were included in this analysis, all of which had very different treatment lengths and intensity (range 5 days – 12 months). Two included participants with mixed eating disorder diagnoses [39, 46] and one included restrictive eating disorder presentations only [45]. Only one MFT study across the lifespan in this review did not find a significant improvement in weight during MFT, however, participants (*N* = 10) in this study reported significant improvements in eating disorder symptoms and mean BMI at the start of treatment was 20.7 (sd = 3.3, range = 16.0–26.1), which was maintained during treatment [45].

Regarding broader psycho-social functioning, there is evidence that MFT is associated with improved quality of life [56, 58], self-perception and self-image [57], self-esteem [58, 59], reduced caregiver burden [54], changes in expressed emotion [60], as well as improved general family functioning [39, 43•, 61, 62] and communication [63]. Notably, change in family functioning and expressed emotion are more varied and not consistently reported between studies.

Within the last few years, data are also beginning to emerge regarding possible moderators, mediators and predictors (baseline individual and family factors) of MFT outcomes. Terache et al. [43•] found that MFT was associated with improvements in all aspects of family functioning on the Family Assessment Device [64], and that two subscales (roles, communication) and the general family functioning score each mediated improvements in some aspects of eating disorder symptomatology measures using the Eating Disorder Inventory, second edition (EDI-II) [65]. Most notably, an improvement in the clarity of family roles mediated changes in drive for thinness [43•]. However, in the same study, family functioning did not mediate improvements in weight [43•]. In another study, decreases in parental perceived isolation was associated with improved young person physical health and general functioning at the end of treatment [54].

Regarding baseline predictors of outcome, Funderud et al. [49•] reported that lower baseline weight was not significantly associated with change in eating disorder symptoms or distress. Dennhag et al. [54] found that baseline maternal level of guilt was associated with poorer end-of-treatment eating disorder symptom outcomes in their case series. Additionally, greater paternal social isolation and perceived burden of dysregulated behaviours was associated with poorer physical health outcomes for the young person at end of treatment [54].

In a secondary analysis of the Eisler et al. [50•] RCT, six hypothesised moderators of treatment effect were recently explored; (1) age, (2) eating disorder symptom severity, (3) perceived conflict from the 3) young person and (4) parent/caregiver perspective, parent-rated (5) positive and (6) negative experiences of caregiving [66]. Positive experiences of caregiving significantly moderated treatment effect. Families presenting with fewer parent-rated positive caregiving experiences at baseline had higher weight at follow-up if they had MFT-AN alongside single-family therapy compared to single-family therapy alone. This is striking given consistent findings from three studies that positive caregiving experiences do not change during MFT [35, 50•, 61]. Taken together, these data suggest that MFT-AN may help protect against this impacting outcome for the adolescent, however, it does not seem help improve parental sense of caregiving itself. The findings from this moderator study extend previous findings that weight at commencement of treatment does not seem to impact on outcome [49•] by demonstrating that it also does not seem to moderate treatment effect [66].

Outcome data for MFT-BN is still very limited, and all data are generated from one outpatient specialist child and adolescent eating disorder clinic, the Maudsley Centre for Child and Adolescent Eating Disorders. Quantitative outcomes (*N* = 50) from this 14-week programme consisting of weekly 90 min sessions indicate it is associated with a reduction in binge-purge behaviours, as well as improvements in eating disorder symptoms, anxiety, depression and emotion dysregulation [48]. Caregiver burden and parental mood also significantly improved in the same study, although level of anxiety in the young person did not [48]. While promising, more data are needed to confirm MFT-BN’s effectiveness and replicability in different contexts.

### Multi-family Therapy Outcomes in Day- and Inpatient Settings

A much smaller amount of data have been reported on for inpatient/day-patient MFT models. Regarding quantitative data, three inpatient (two young person [61, 67] and one adult [35] studies) and one adult day programme MFT study [44] have been published. All studies compared MFT to another type of treatment, namely, single family therapy [35, 44, 67] or a parent-only group intervention [61]. Two were small RCTs (*N* = 25 [67], *N* = 48 [35]) and the other two used a non-randomised, uncontrolled comparison design.

Regarding outcomes, MFT within intensive treatment settings has been associated with weight gain and eating disorder symptom improvement [35, 44, 61], as well as broader factors such as expressed emotion [35, 44], perceived caregiver burden [61], family member depressive symptoms [44], carer distress [35] and family functioning [61].

Notably, findings are more mixed than in the outpatient context. One study found no change in eating disorder symptoms and a worsening in perceived family functioning, which the authors attributed to increased family insight into their difficulties [67]. This may reflect the more heterogeneous treatment models tested and the fact that much more intervention is offered alongside MFT in inpatient and day-patient settings, such as other therapeutic group work, meal support, individual and family therapy sessions, etc.

### Qualitative Findings and Perceived Change Processes During MFT

Due to the multiple co-occurring family needs and dynamics within MFT, increasingly qualitative studies are being produced to better understand participant, caregiver and clinician experiences of treatment. Based on data generated using observational, individual interviews and focus groups, MFT has been described as challenging and helpful, regardless of model, setting or age [1]. Specifically, people describe it as promoting understanding, identity development, mentalising and holistic recovery-oriented change [47, 63, 68–71]. Benefits include participants (particularly caregivers) feeling empowered, more confident and more able to share experiences [45, 72–74]. Many also speak of the intensity and comparisons that arise out of the group process, and how this can generate and lead to the expression of strong emotion – described as both a challenge and a benefit [62, 68]. Being able to learn and experiment through non-verbal and activity-based tasks has also been described as helpful and unique to MFT [68].

In the only study exploring the experience of MFT delivered online vs. face-to-face, data were mixed with participants describing both the convenience of online MFT as well as the potential loss of non-verbal communication during online working [62].

**Fig. 1:**
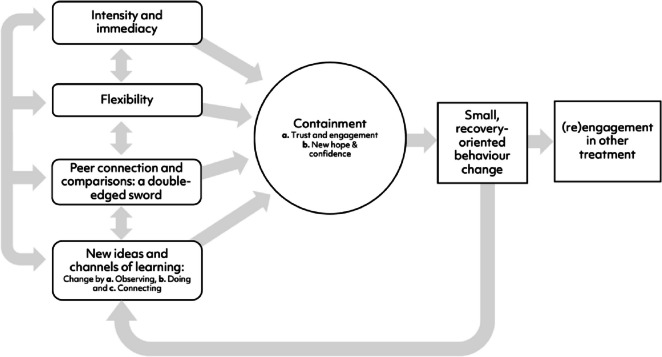
Proposed model of change in MFT-AN that incorporates young person, parent/caregiver and clinician perspectives from two recent qualitative studies [68, 75]

Most recently, qualitative studies have explored how participants and clinicians perceived change to occur within MFT [68, 75]. Considering data from these three different perspectives suggests four inter-related elements (1. Intensity and immediacy, 2. Flexibility, 3. Peer connection and comparisons, 4. New ideas and channels of learning) may combine to contain the family system by building trust, engagement, hope and confidence. It seems that from this (re)established secure base, recovery-oriented behaviour change may (re)commence. See Fig. 1 for a proposed model of change in MFT-AN that incorporates data from clinician [75], young person and parent/caregiver [68] data. To date, no data have been collected regarding potential change processes in MFT-BN.

Within this model (Fig. 1), the step of ‘containment’ may reflect the quantitative findings that improved family functioning mediates MFT outcomes [43•]. Qualitative data suggests families need to move from a place of uncertainty and distress to connection to be able to take positive steps forward. This fits closely theoretically with the aspects of family functioning (roles, communication, general family functioning) that mediated outcomes in the Terache et al. [43•] quantitative study. It also supports the idea that MFT (and possibly other intensive treatments) may (re)activate several change processes recently reported for single-family therapy [76].

These data also fit theoretically with qualitative data on young person and parent experiences of intensive day programme treatment [77, 78]. While, day programme treatments are somewhat different from MFT, some do include multi-family elements. Other similarities include the intensity, increased level of support and additional hours of intervention per week. In both settings, the importance of immediacy, intensity and connecting with others are all described by young people and parents/caregivers as key factors in supporting people to (re)engage in less intensive outpatient treatment.

## Conclusions

Available data are encouraging and suggest MFT is an effective adjunctive treatment for people with eating disorders across the lifespan. The available data are predominantly focused on adolescent MFT-AN models, specifically. Conclusions are more mixed regarding adult MFT models and MFT-BN. Findings for this review indicate that more is needed to confirm these effects in more diverse and larger groups, and settings. It will be important for future research to begin empirically investigating which components or aspects of MFT are most effective and whether this differs according to individual, family, cultural and service contexts.

## Data Availability

No datasets were generated or analysed during the current study.
